# Association of COVID-19 Quarantine Duration and Postquarantine Transmission Risk in 4 University Cohorts

**DOI:** 10.1001/jamanetworkopen.2022.0088

**Published:** 2022-02-25

**Authors:** Andrew Bo Liu, Dan Davidi, Hannah Emily Landsberg, Maria Francesconi, Judy T. Platt, Giang T. Nguyen, Sehyo Yune, Anastasia Deckard, Jamie Puglin, Steven B. Haase, Davidson H. Hamer, Michael Springer

**Affiliations:** 1 Bioinformatics and Integrative Genomics PhD Program, Harvard Medical School, Boston, Massachusetts; 2 Department of Genetics, Harvard Medical School, Boston, Massachusetts; 3 Student Health Services, Boston University, Boston, Massachusetts; 4 Harvard University Health Services, Boston, Massachusetts; 5 Department of Medicine, Harvard Medical School, Boston, Massachusetts; 6 Student Affairs Northeastern University, Boston, Massachusetts; 7 Office of Information Technology, Duke University, Durham, North Carolina; 8 Office of Assessment, Duke University, Durham, North Carolina; 9 Department of Medicine, Duke University School of Medicine, Durham, North Carolina; 10 Department of Global Health, Boston University School of Public Health, Boston, Massachusetts; 11 Section of Infectious Diseases, Department of Medicine, Boston University School of Medicine, Boston, Massachusetts; 12 National Emerging Infectious Diseases Laboratory, Boston University, Boston, Massachusetts; 13 Department of Systems Biology, Harvard Medical School, Boston, Massachusetts

## Abstract

**Importance:**

Optimal quarantine length for COVID-19 infection is unclear, in part owing to limited empirical data.

**Objective:**

To assess postquarantine transmission risk for various quarantine lengths and potential associations between quarantine strictness and transmission risk.

**Design, Setting, and Participants:**

Retrospective cohort study in 4 US universities from September 2020 to February 2021, including 3641 university students and staff who were identified as close contacts to individuals who tested positive for SARS-CoV-2 infection. Individuals were tested throughout the 10 to 14–day quarantine, and follow-up testing continued at least weekly throughout the 2020-2021 academic year.

**Exposures:**

Strict quarantine, including designated housing with a private room, private bathroom, and meal delivery, vs nonstrict, which potentially included interactions with household members.

**Main Outcomes and Measures:**

Dates of last known exposure, last negative test result, and first positive test result during quarantine.

**Results:**

This study included 301 quarantined university students and staff who tested SARS-CoV-2–positive (of 3641 quarantined total). These 301 individuals had a median (IQR) age of 22.0 (20.0-25.0) years; 131 (43.5%) identified as female; and 20 (6.6%) were staff. Of the 287 self-reporting race and ethnicity according to university-defined classifications, 21 (7.3%) were African American or Black, 60 (20.9%) Asian, 17 (5.9%) Hispanic or Latinx, 174 (60.6%) White, and 15 (5.2%) other (including multiracial and/or multiethnic). Of the 301 participants, 40 (13.3%; 95% CI, 9.9%-17.6%) had negative test results and were asymptomatic on day 7 compared with 15 (4.9%; 95% CI, 3.0%-8.1%) and 4 (1.4%; 95% CI, 0.4%-3.5%) on days 10 and 14, respectively. Individuals in strict quarantine tested positive less frequently than those in nonstrict quarantine (10% vs 12%; *P* = .04).

**Conclusions and Relevance:**

To maintain the 5% transmission risk used as the basis for US Centers for Disease Control and Prevention’s 7-day test-based quarantine guidance, our data suggest that quarantine with quantitative polymerase chain reaction testing 1 day before intended release should be 10 days for nonstrict quarantine and 8 days for strict quarantine, as ongoing exposure during quarantine may be associated with the higher rate of positive test results following nonstrict quarantine.

## Key Points

**Question:**

What is the risk of SARS-CoV-2 transmission from individuals leaving test-based quarantines of various durations?

**Findings:**

In this cohort study of 301 quarantined university students and staff who tested positive for COVID-19, 40 (13.3%) tested negative and were asymptomatic on day 7, implying an approximate 13% postquarantine transmission risk for 7-day test-based quarantine.

**Meaning:**

To maintain the 5% transmission risk used as the basis for the 7-day guideline, our data suggest that quantitative polymerase chain reaction test–based, nonstrict quarantine should be 10 days.

## Teaser

This cohort study evaluates transmission risk following quarantine periods of varying lengths and degrees of strictness among US university students and staff.

## Introduction

COVID-19, caused by SARS-CoV-2, has caused an unprecedented global public health crisis.^1^ Isolating infected individuals and identifying and quarantining their close contacts remain key strategies used to mitigate the spread of SARS-CoV-2. Quarantine is used to separate individuals who might have been exposed to SARS-CoV-2 to minimize their risk of transmitting SARS-CoV-2 to other people. Most of the world remains unvaccinated against COVID-19, and quarantine is still being used even in countries with relatively high vaccination rates.

Quarantine length is a balance: a short quarantine brings increased risk of transmission from individuals who are infectious after release, while a long one may increase transmission risk by reducing compliance, stretching public health systems, and imposing additional economic and psychological hardship.^2^ The US Centers for Disease Control and Prevention (CDC) initially recommended a 14-day quarantine period based on estimates of the upper bound of the SARS-CoV-2 incubation period.^2,3^ While the CDC still recommends 14-day quarantine as the preferred option, in November 2020, to account for the costs of long quarantine, the organization identified 2 shorter quarantine options as acceptable alternatives for asymptomatic individuals based on local circumstances and resources. The first was a 10-day quarantine period without testing. The second was a 7-day quarantine if results of a test done on day 5, 6, or 7 were negative.^2^ Similarly, France instituted a 7-day quarantine and Belgium, Germany, and Spain adopted a 10-day period^4^; and, on the longer end, Chinese cities Beijing and Dalian adopted a 21-day quarantine period.^5^

Knowing more about the dynamics of SARS-CoV-2 infection can improve assessments of quarantine duration guidelines. If we know that most people convert by reverse transcriptase–quantitative polymerase chain reaction (RT-qPCR) test before a certain day postexposure, individuals with negative test results on that day are unlikely to be SARS-CoV-2–positive afterwards. A previous study measured SARS-CoV-2 positivity at different times in quarantined students in kindergarten through 12th grade; however, it was difficult to draw strong conclusions from this study as positive test results were few and students were tested infrequently, and rarely before day 7 following exposure.^6^

Here we report the conversion times—the times between exposure and testing positive for SARS-CoV-2—for 301 unvaccinated university students and staff who were identified as close contacts with individuals with COVID-19 infection, quarantined, and later tested positive between September 2020 and February 2021. Transmission from test-based quarantine, where people are released from quarantine based on negative RT-qPCR results, occurs when individuals first test positive after release. These individuals spend their full qPCR-positive period and most of their infectious period outside quarantine. Thus we can estimate transmission and set quarantine duration based on these conversion times (for non–test-based quarantine, metrics like latent period are more useful^7^).

## Methods

Our research protocol was approved by the institutional review board of the Harvard Faculty of Medicine, and we have data use agreements with Boston University, Duke University, and Northeastern University. Written consent was obtained by each university as part of their university testing program. The written consent allowed for broad use of the deidentified and aggregated data for public health and research purposes. We followed the Strengthening the Reporting of Observational Studies in Epidemiology (STROBE) guidelines for reporting study design, follow-up duration, participant recruitment methods, and other observational study details.

**Figure 1.**
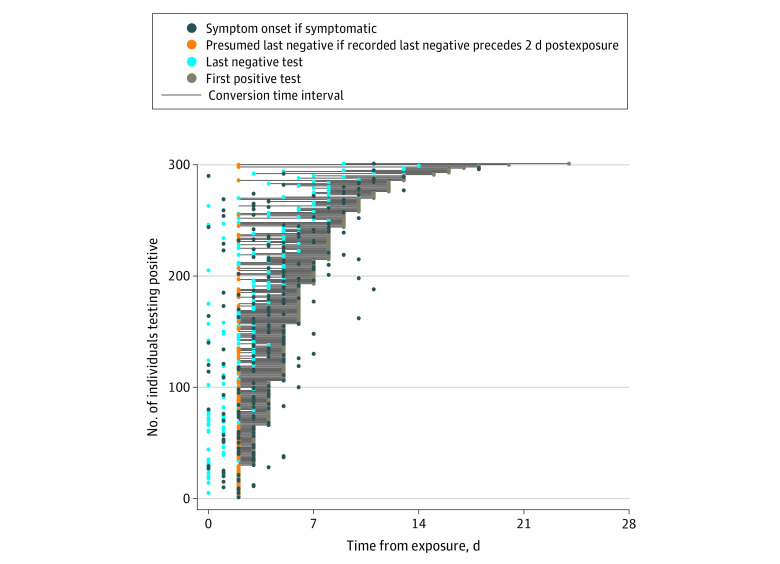
Conversion Time Intervals of 301 Quarantined University Students and Staff With Positive Test Results

Four universities (Boston University, Boston, Massachusetts; Duke University, Durham, North Carolina; Harvard University, Boston, Massachusetts; and Northeastern University, Boston, Massachusetts) reported data from 418 students and staff who were quarantined because of potential SARS-CoV-2 exposure and subsequently tested positive between September 2020 and February 2021. Of 418 individuals with positive test results, 301 had complete data on presence of symptoms, date of last exposure, last negative test result during quarantine, first positive test result during quarantine, and date of symptom onset if symptoms occurred. These 301 individuals were included in our study. These data enable us to place the conversion time in the interval between the last negative test result and first positive test result. A total of 117 individuals with a first RT-PCR positive test by day 2 following exposure were excluded from the study, as it was assumed that these individuals were infected at an earlier date. We set the last negative date at 2 days postexposure if the recorded last negative date was before then, given that individuals with SARS-CoV-2 infection have not typically been observed to receive positive test results until at least 2 days postexposure.^8,9^ Conversion times are reported in terms of days after exposure. The universities’ frequent testing enabled us to pinpoint conversion time to within at most a 4-day interval in 84% of cases (254 of 301 individuals) (Figure 1).

The contact tracing protocols in all 4 universities were based on CDC and local public health guidelines. A total of 3641 students and staff were identified as close contacts and entered quarantine; if they lived on campus, they entered strict quarantine, which included housing specifically designated for quarantine, with meal delivery, linens, and self-care necessities provided. All staff with positive test results quarantined off campus and were in nonstrict quarantine. Testing was generally conducted twice per week, with minor variation in testing frequency between universities and between individuals. If during contact tracing interviews it was discovered that an individual with positive test results had recently been released from quarantine, tracing teams followed up by asking about potential reexposure either during or after quarantine. Any instance of cohabitation with an infected individual was deemed a possible reexposure. The detailed testing, contact tracing, and quarantine protocols can be found in eMethods in the Supplement. A total of 227 of 301 individuals (75%) developed symptoms (186 [62%] before day 7 and 211 [70%] before day 10), and the remaining 74 (25%) were asymptomatic.

Each university used different PCR test protocols (eMethods in the Supplement). One university used viral gene target E; 2 used targets N1 and N2, requiring at least 1 to have a cycle threshold value of 40 or less (along with ribonuclease P as a human material control); and 1 used targets ORF1a, ORF1b, and S, requiring at least 2 of the 3 to have cycle threshold values of 35 or less. Three universities reported using in-house testing and 1 reported using the Thermo Fisher TaqPath multiplex commercial kit. Further details about the performance and protocol of each assay can be found as part of each university’s emergency use authorization.^10,11,12,13,14^

Participant race and ethnicity classifications were self-reported, typically in student records. Race and ethnicity classifications options were typically defined by the university. Race and ethnicity were not assessed in this study.

### Statistical Analysis

We estimated the percentage of conversion on each day, and then estimated transmission risk for a quarantine protocol that included symptom attestation and a negative test result by aggregating smoothed uncertainty intervals of conversion times for the 301 individuals with positive test results and complete data (eMethods in the Supplement). We assumed real conversion times were more likely in the center of the interval than in the periphery. Results were similar if we assumed conversion times were equally likely across the whole interval (eFigure in the Supplement). We estimated 95% CIs based on Agresti-Coull and used the PropCIs package (R version 4.1.2 [R Project for Statistical Computing]) for calculation.

**Figure 2.**
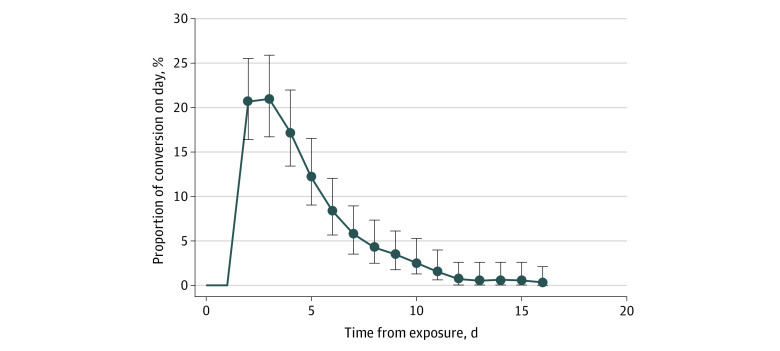
Determining Conversion Times of 301 University Students and Staff Line shows the probability distribution of conversion times. Error bars represent 95% CIs. Conversion values after day 16 sum to 0.3%.

**Figure 3.**
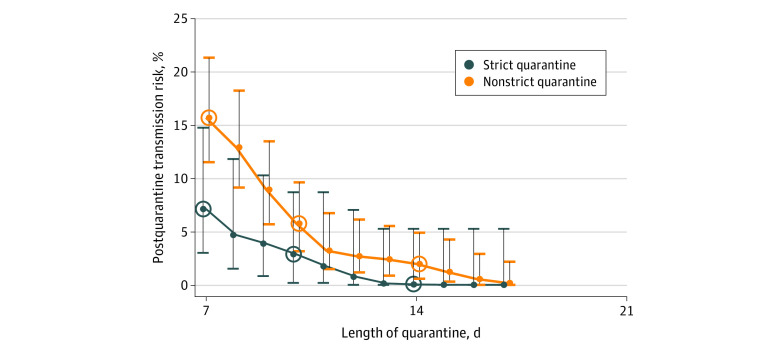
Transmission Risk After Release From Test-Based Strict and Nonstrict Quarantines of Various Lengths Individuals were released from quarantine if they received a negative test result and were asymptomatic; thus risk is the percentage of individuals with positive test results who first received positive test results after the given day and were asymptomatic until that day. Plots show transmission risk based on conversions for the positive test results of 301 university students and staff. Values are shown for days 7 (corresponding to the US Centers for Disease Control and Prevention [CDC] quarantine with testing alternative guideline), 10 (corresponding to the CDC quarantine without testing alternative guideline), and 14 (corresponding to the original CDC quarantine guideline). Strict quarantine included designated housing that consisted of a private room, private bathroom, and meal delivery. Nonstrict quarantine potentially included interactions with other household members. Error bars represent 95% CIs.

Our estimates do not account for false-negative results because they were rare in this study population. While false-negative results do occur, the rate of false-negative results in this study was likely lower than 5%, as evidenced by (1) 98.6% coherence between positive test results from individuals who used 2 independent swabs (72 of 73 individuals) and (2) the fact that individuals with confirmed positive results who were tested daily did not have a negative result until late in their recovery phase. Even if our false-negative rate were 5%, it would not change our transmission estimates substantially; for example, our 13.3% post–day 7 transmission estimate becomes 12.7% if we assume 5% of last negative test results were truly positive. We did not separately analyze the staff because their low sample size (20 of 301 individuals) made it difficult to make a statistically meaningful comparison with the students. The 2-sided proportion test comparing conversion rates in strict and nonstrict quarantine was calculated using R version 4.1.2 (the R Foundation) function at a 95% confidence level with the Yates continuity correction, and significance was assessed at *P* = .05. Note that the percentages of conversion in Figure 2 are slightly higher than the transmission risks in Figure 3. This difference is owing to the fact that some people develop symptoms before receiving a positive RT-PCR test result; these individuals, per the CDC alternative guideline, are required to complete a full 14-day quarantine.

## Results

These 301 individuals included in this study had a median (IQR) age of 22.0 (20.0-25.0) years; 131 individuals (43.5%) identified as female; and 20 individuals (6.6%) were staff. Of the 287 individuals who reported race and ethnicity, 21 individuals (7.3%) were African American or Black, 60 (20.9%) Asian, 17 (5.9%) Hispanic or Latinx, 174 (60.6%) White, and 15 (5.2%) other (including multiracial and/or multiethnic). We analyzed conversion times for these 301 individuals (Figure 1), as measured from the last exposure date to the dates of the last negative and first positive test results. These are aggregated into a graph of conversion time by day (Figure 2).

We estimated the risk of conversion after release from quarantine by aggregating conversion time (Figure 3). In strict and nonstrict quarantine combined, 40 of 301 individuals with positive test results (13.3%; 95% CI, 9.9%-17.6%) were asymptomatic and had not yet converted on day 7. Under the 7-day guideline, these 40 would have been released on day 7 and converted afterwards, reflecting postquarantine conversion and therefore transmission risk. Similarly, of 301 individuals who converted, 15 (4.9%; 95% CI 3.0%-8.1%) and 4 (1.4%; 95% CI, 0.4%-3.5%) had negative test results and were asymptomatic on days 10 and 14, respectively.

Stricter quarantine was associated with shorter conversion times. In strict quarantine, 6 (7.1%; 95% CI, 3.0%-15.0%), 2 (2.9%; 95% CI, 0.2%-8.7%), and 0 (0%; 95% CI, 0%-5.3%) individuals who converted did so after days 7, 10, and 14, respectively, compared with 34 (16.0%; 95% CI, 12.0%-21.0%), 12 (5.8%; 95% CI, 3.2%-9.6%), and 4 (2.0%; 95% CI, 0.6%-4.9%) in nonstrict quarantine. In 5 of the post–day 10 individuals in nonstrict quarantine who converted, additional questioning by contact tracers revealed that, in all 5 cases, the individual was reexposed to a person with COVID-19 during their quarantine. This suggests that a higher rate of repeated exposures during quarantine may explain the longer conversion times seen in nonstrict quarantine; thus, strict quarantine data are more likely to provide accurate estimates of conversion times in quarantine.

Of 1319 exposed individuals in strict quarantine, 132 (10%) converted; of 2322 exposed individuals in nonstrict quarantine, 286 (12%) converted. Thus, stricter quarantine was associated with a lower chance of getting a positive test result (10% vs 12%; *P* = .04 in 2-sided proportion test) suggesting that nonstrict quarantine could contain individuals who were reexposed during quarantine. This result, along with shorter conversion times in stricter quarantine, suggests that stricter quarantine is associated with reduced transmission.

## Discussion

In this study, in 301 quarantined, positive-testing university students and staff, 40 (13.3%) first tested positive asymptomatically after day 7 following exposure, 15 (4.9%) after day 10, and 4 (1.4%) after day 14. Individuals in strict quarantine tested positive at a lower rate than those in nonstrict quarantine (10% vs 12%; *P* = .04).

The 13.3% post–day 7 conversion rate implies that 13% of transmission may occur after release from 7-day quarantine. The CDC mathematically estimated this post–7-day quarantine transmission risk as 2.3% to 8.6%^2^ for strict quarantine only, whereas our 13% estimate is for combined strict and nonstrict quarantine. Most household or hotel quarantines probably more closely resemble nonstrict than strict quarantine.^15,16^ Thus we argue that the 13% estimate is generally more relevant for determining quarantine length, although jurisdictions should focus on the estimates most relevant to their quarantine strictness. To limit postquarantine transmission risk to 5% of total risk in a pre–Alpha variant university setting, our data suggest that quarantine with RT-qPCR testing 1 day before intended release would need to extend to 10 days for nonstrict quarantine and 8 days for strict quarantine. To achieve a postquarantine transmission risk lower than 5% or to achieve 5% in settings without testing would require even longer quarantines (Figure 3).

As noted in the CDC guidance, quarantine is “intended to physically separate a person exposed to COVID-19 from others.”^2^ While both nonstrict and strict quarantine intended to implement this separation, strict quarantine was associated with lower transmission risk in our university cohorts. Our study suggests that a substantial number of people may be getting reexposed during quarantine. A strict shorter quarantine may be as effective as a longer more lax one.

### Limitations

This study has limitations. Quarantine policies need to take into account many factors, including socioeconomic cost, mental health, and the effectiveness of contact tracing. For example, the overall conversion rate in a given setting depends on the effectiveness of contact tracing and the stringency of guidelines for determining close contacts. Hence, jurisdictions should consider our results as one factor among several in their overall quarantine guidance. They should also consider that longer quarantines may reduce willingness to comply or impose economic hardships, which reduce ability to quarantine. It should be noted that our data are from unvaccinated individuals and are biased toward young individuals with high-socioeconomic status in private universities.

We expect our findings to hold for all the current variants of concern. A variant will mainly change transmission from test-based quarantine if its latent period is longer; there is currently no evidence for this. Recent data show the incubation period, which should correlate with latent period,^7^ is largely unchanged in the new variants. In particular, Public Health England tracked almost 7000 cases of transmission of Alpha and Delta variants and found that median incubation periods were 4 and 4 to 5 days, respectively.^17^ Table 8 in their publication presents these incubation periods, which are similar to the previously reported 5- to 6-day incubation periods for COVID-19 in 2020.^7^

## Conclusions

Our results provide evidence of the risk of transmission from people released from test-based quarantine. Individuals in strict quarantine tested positive at a lower rate than those in nonstrict quarantine. This study shows the importance of empirical validation of quarantine guidelines and offers an approach for such validation. As future variants emerge, similar analyses should be conducted to ensure the guidance remains relevant.

## Notes

Supplement.
eMethods.
**eFigure. **Post-Quarantine Transmission Risk for Individuals in Strict and Nonstrict Quarantine Based on Uniform Kernel
